# Correlation of Simulation Examination to Written Test Scores for Advanced Cardiac Life Support Testing: Prospective Cohort Study

**DOI:** 10.5811/westjem.2015.10.26974

**Published:** 2015-11-22

**Authors:** Suzanne L. Strom, Craig L. Anderson, Luanna Yang, Cecilia Canales, Alpesh Amin, Shahram Lotfipour, C. Eric McCoy, Mark I. Langdorf

**Affiliations:** * University of California Irvine School of Medicine, Department of Anesthesia and Perioperative Care, Irvine, California; † University of California Irvine School of Medicine, Department of Emergency; ‡ Medicine, Irvine, California; § University of California Irvine, Irvine, CaliforniaUniversity of California Irvine School of Medicine, Department of Medicine, Irvine, California

## Abstract

**Introduction:**

Traditional Advanced Cardiac Life Support (ACLS) courses are evaluated using written multiple-choice tests. High-fidelity simulation is a widely used adjunct to didactic content, and has been used in many specialties as a training resource as well as an evaluative tool. There are no data to our knowledge that compare simulation examination scores with written test scores for ACLS courses.

**Objective:**

To compare and correlate a novel high-fidelity simulation-based evaluation with traditional written testing for senior medical students in an ACLS course.

**Methods:**

We performed a prospective cohort study to determine the correlation between simulation-based evaluation and traditional written testing in a medical school simulation center. Students were tested on a standard acute coronary syndrome/ventricular fibrillation cardiac arrest scenario. Our primary outcome measure was correlation of exam results for 19 volunteer fourth-year medical students after a 32-hour ACLS-based Resuscitation Boot Camp course. Our secondary outcome was comparison of simulation-based vs. written outcome scores.

**Results:**

The composite average score on the written evaluation was substantially higher (93.6%) than the simulation performance score (81.3%, absolute difference 12.3%, 95% CI [10.6–14.0%], p<0.00005). We found a statistically significant moderate correlation between simulation scenario test performance and traditional written testing (Pearson r=0.48, p=0.04), validating the new evaluation method.

**Conclusion:**

Simulation-based ACLS evaluation methods correlate with traditional written testing and demonstrate resuscitation knowledge and skills. Simulation may be a more discriminating and challenging testing method, as students scored higher on written evaluation methods compared to simulation.

## INTRODUCTION

There is early and promising evidence that high-fidelity simulation may be more effective in training healthcare providers in the management of critically ill patients.^1^–^4^ Previous work has reported its use to assess the psychomotor performance of senior medical students on the American Heart Association’s (AHA) standardized Advanced Cardiac Life Support (ACLS) clinical resuscitation scenarios.^5^ This research showed that a simulation-based course in ACLS resulted in enhanced student performance, with improved critical action completion, clinical knowledge and psychomotor skill application, and decreased time to cardiopulmonary resuscitation (CPR) and defibrillation.

Student assessment of knowledge acquisition after an ACLS course is traditionally performed using multiple-choice testing alone, with practical skills demonstration of basic airway management, CPR and defibrillation. Although with little evidence to support its use, written evaluations for the assessment of critical management skills has been the historical standard. The advent of evidenced-based medicine and medical simulation has created debate on the optimal evaluation method to assess medical students’ ability to manage critically ill patients.

We are not aware of any literature that evaluates the relationship between integrated high-fidelity simulation-based methods and traditional written cognitive testing with non-integrated psychomotor performance.^6^ This evaluation was recommended as one of the critical steps of core competency assessment by a professional academic society working group on assessment of observable learner performance.

The objective of our study was to correlate results of a novel high-fidelity simulation-based evaluation method with traditional written evaluation for senior medical students enrolled in an ACLS course.

## METHODS

We performed a prospective cohort study evaluating the correlation between high-fidelity simulation-based evaluation with traditional written testing for senior medical students enrolled in an ACLS course. The study was conducted in a medical school simulation center. We obtained institutional review board approval to record simulation sessions and collect patient management data from 19 student volunteers (11 females), most interested in careers in emergency medicine, anesthesiology, or surgery. The course was held over a four-day period in one school week in the last quarter of the senior year. We recorded each student managing a standard acute coronary syndrome (ACS)/ventricular fibrillation (VF) cardiac arrest scenario just prior to the start of the course, and then tested them in both written and simulation format (identical cardiac arrest scenario) upon completion of the course. The 32-hour course consisted of 12 hours of didactics, eight hours of simulation training, eight hours of self-study time, and four hours of post-course practical and written testing.

The three traditional written evaluation instruments were the following: 1) a multiple-choice test, 2) a cardiac rhythm test, and 3) a clinical management test. The 36 questions of multiple choice were developed by the AHA, which covered the breadth of content from the ACLS Student Manual. The questions focused on basic and advanced airway management, algorithm application, resuscitative pharmacology, and special situations like drowning and stroke recognition. The rhythm knowledge evaluation consisted of 20 examples of various brady-and tachyarrhythmias, heart blocks and asystole/agonal rhythm to which the students were required to match rhythm diagnoses on a one-to-one basis. The clinical management “therapeutic modalities” was a fill-in-the-blank test including seven clinical scenarios: ACS, symptomatic bradycardia, pulseless electrical activity, refractory VF, stable and then unstable ventricular tachycardia, third-degree heart block, and asystole (appendices 1, 2 and 3). All written evaluation tools were based on content from the ACLS student manual or obtained from the AHA. All testing protocols and tools were evaluated by two expert ACLS instructors/experienced clinicians (anesthesiologist and emergency physician) prior to implementation of the course. Although we weighted the three components equally in the composite “correct answer” score, the maximum possible written test points were 36 (multiple choice), 20 (rhythms) and 61 (“therapeutic modalities”).

To assess post-course ACLS skills, students directed a high-fidelity simulation scenario of a patient with ST-elevation acute myocardial infarction, VF cardiac arrest, defibrillation, basic and advanced airway management, return of spontaneous circulation (ROSC), third-degree heart block, hypotension, acidosis and activation of the cardiac catheterization team. The simulation-based assessment was clinically oriented and approximated the course of events that would take place in the management of a real patient. Each student was tested without additional team members to whom they would normally delegate tasks.

We judged resuscitation successful and awarded ROSC if the student began near-continuous CPR, performed effective bag-valve-mask and/or endotracheal intubation, defibrillated with appropriate joules, and administered two correct doses of epinephrine (or one of vasopressin) and either lidocaine or amiodarone in appropriate doses. We calculated the Kappa statistic for inter-rater reliability. Disagreements in scoring were resolved by jointly reviewing the videos.

Students performed their simulations in a state-of-the-art simulation center approximating a resuscitation room in a modern emergency department. The equipment used in the 65,000+ square-foot medical simulation center included a SimMan 3G © (Laerdal, Wappinger Falls, NY), live defibrillator and crash cart, cardiac monitor, and basic and advanced airway equipment. We used B-line Medical Simbridge software © (B-line Medical, Washington, DC) for video capture, storage and review.

A technical skills checklist of critical actions for the scenarios was created by clinical and simulation faculty using a modified-Delphi technique. Prior to participation in the ACLS course, subjects were recorded performing as team leader in the standard simulation scenario. The students then completed the Resuscitation Boot Camp with imbedded ACLS course and, as a final test, each student was recorded repeating the same ACS/VF scenario (12–15 minutes). Two expert ACLS instructors (one a regional faculty member) scored the recordings of the before and after performances separately on a 121-point scale and the mean of their assessments was used for analysis. To foster inter-rater reliability, the two instructors jointly developed the scoring scheme, identified each action item, agreed to meaning of the description of the action, and assigned point values. The instructors were not blinded to the study hypothesis, but were blinded to the students’ written test performance.

Our primary outcome measure was the correlation between the simulation-based evaluation method and the traditional written evaluation. Our secondary outcome was the comparison of the two scores between the modalities.

We excluded one student who scored very poorly on the written test component of cardiac rhythm interpretation at 55% correct. All other students scored 90–100% on this testing modality. The excluded student’s overall score was 78.0% correct, while all other students scored means of 86.6–98.1% correct. Therefore, the excluded student was a clear outlier.

We used t-tests for paired data to compare written and simulation test scores, with each student serving as their own control. We used linear regression to quantify the relationship between the two sets of scores, and set statistical significance at p<0.05. (STATA version 12.1, StataCorp, College Station, Texas)

## RESULTS

The composite average score on the three written evaluations was substantially higher (93.6%) than the simulation performance score (81.3%, absolute difference 12.3%, 95% CI [10.6–14.0%], p<0.00005). The various component mean and SD scores are listed in the Table.

We found a statistically significant moderate correlation between simulation scenario test performance and traditional written test performance (Figure) (Pearson r=0.48, p=0.04).

Inter-rater reliability for scoring the participants in pre- and post- training scenarios was good. The median kappa for the 75 test items was 0.68 (interquartile range 0.36–0.94). Forty-six items (61%) had kappa >0.60.

## DISCUSSION

We found that high-fidelity simulation-based evaluation and traditional written testing for senior medical students in an ACLS course correlates well with each other. Simulation is being incorporated in the education, training, and evaluation of healthcare providers at a rapid pace. As educational technology advances rapidly, the research to support its use has lagged behind. Traditional written evaluations are widely used, and have been accepted as the standard for healthcare providers’ ability to manage critical patients. However, as simulation is realistic, actively engaging and clinically based, healthcare teachers have begun to question written testing.^7^

We found a positive moderate correlation between simulation-based evaluation and traditional written evaluation. Other studies have compared the two but did not specifically assess correlation, nor report results in medical trainees. Rodgers’ study on nursing students in an ACLS course completing both a written and practical evaluation concluded that written evaluation is, not surprisingly, a poor predictor of skill performance.^8^ Issenberg similarly found no association between CPR psychomotor skills and total knowledge in nursing students.^9^ As physician trainees are destined to be team leaders in resuscitation, our work is the first to study medical students, and therefore adds to this literature.^10^,^11^

The issue of correlation should not be misconstrued as equivalence. We contend that the simulation evaluation is superior to evaluate psychomotor skills, yet accept the place of written evaluation to demonstrate cognitive mastery across broad medical content. The correlation demonstrates that students who have traditionally done well in written testing are likely to also do well in a simulation evaluation. An educator should acknowledge that the two evaluation methods are complementary, rather than substitutable, and consider adding such evaluation to tasks that require manual dexterity and critical thinking. Furthermore, simulation requires substantial human and capital resources to show competence, and therefore limits its widespread application. Simulation, by necessity, focuses on narrow clinical scenarios, which, though chosen to represent critical management, cannot cover the entire breadth of cardiac resuscitation. Hence both simulation and written evaluations are likely necessary.

Our secondary outcome compared the two scores. The composite average score on the written evaluation was substantially higher (93.6%) than the simulation performance score (81.3%). It is important to note that all 19 students had the same training and were evaluated by both written and simulation methods. A higher written test score does not mean better performance, as the two modalities measure different outcomes.

Participants find high-fidelity simulation for critical event management to be a valuable educational experience.^12^ Emotional arousal is effective in memory acquisition^13^ and simulation-based experiential learning has been shown to be effective in retention of skills,^14^ improving clinical outcomes,^15^ and reducing error related healthcare costs.^16^ Furthermore, repetition of simulation experience reinforces knowledge acquisition and increases confidence.^17^,^18^

Written testing has historically been the most common mode of evaluation. However, the construct validity of the AHA’s ACLS test has been challenged, as nurses’ scores were not shown to correlate with performance on resuscitation after an ACLS course. These same authors opined that the written testing at least had content validity, as the tests questions were drawn directly from the student manual. Finally, their analysis supported our contention that the two modalities complement each other in providing a broad assessment of the learner’s performance.^19^

Despite these questions, newer examination techniques, such as simulation-based evaluations, need to be validated before widespread use. Our study provides preliminary evidence that will shape this discussion. There has been a move toward simulation for assessment, as exemplified by a report of five years of certification via Fundamentals of Laparoscopic Surgery.^20^ In addition, simulation is used in both initial and maintenance of certification in anesthesiology.^21^–^23^ Hence, it is critical to scrutinize new testing methods to validate that they at least approximate traditional techniques.

Students performed better on the written form of testing than on the simulation. We believe this indicates that the simulation evaluation method is a more demanding measure, which emphasizes application of knowledge over rote memorization. Furthermore, we found a narrower range of student performance with the simulation method (range of scores 74.8–87.2%, Δ12.4%) than the written assessment (80.9–98.2%, Δ17.3%), which indicates a more uniform and direct performance in concert with course goals. Since the purpose of the boot camp is to prepare students for clinical practice, an instrument/method that better generates a consistent execution of skills is valued over abstract knowledge applied in isolation. In the end, the educator should consider using both methods of evaluation when teaching psychomotor skills. In addition, a quantitative simulation evaluation with an established “pass” threshold should be incorporated, in order to move toward competency-based evaluation.

## LIMITATIONS

Our study has limitations, including enrolling a small sample of self-selected, highly motivated students entering the fields of emergency medicine, anesthesiology, or surgery. We did not have any baseline data on the subjects’ prior ACLS training or experience. However, this did not affect our study’s ability to evaluate the relationship between a simulation-based and written evaluation tool, as students served as their own controls. We used a previously non-validated simulation evaluation scale with arbitrary weighting of points for critical actions (derived from two expert ACLS instructors), though the action items had been used for grading in the course for 15 years. Furthermore, our assessment tool is based on AHA guidelines, and is clinically focused on critical action items that approximate real clinical care, compared to a multiple-choice or even fill-in-the-blank format. Our course was non-traditional and expanded from ACLS, and included advanced airway management and additional didactics. However, both assessment methods tested knowledge and skills from this non-traditional course format, which would not confound the assessment methods themselves. Our criterion reference was the ACLS written exam. To our knowledge, these test questions are not analyzed for reliability or validity. There are no previous studies that demonstrate construct validity of the AHA written examination or correlate clinical performance with the written examination. The correlation between written and simulation examination performance in this study does demonstrate some degree of construct validity. The written examination is based entirely on the ACLS manual and should therefore have content validity.

To provide maximal experience with simulation and to reinforce specific and detailed proper ACS/cardiac arrest management, we used the same teaching and testing scenario and informed the students that the pre- and post-tests would be identical. This may have artificially improved post-test performance through studying specifically for the known test, as well as additional familiarity with the simulation technology. We did not control for progressive experience and therefore comfort with the mannequin or simulation experience, nor was there a traditional ACLS course student control group.

Future studies should use students destined for all specialty residencies, and assess the rate of long-term retention of psychomotor skills.

We excluded one outlier who scored far below the other students, at 55% correct on the rhythm matching test (11/20 correct). This student scored average on the simulation evaluation, which only required identification of three (not 20) obvious rhythms. Including this outlier would have made our correlation fall short of statistical significance. However, the scatter plot visually demonstrates the conclusion that higher written scores are associated with higher simulation scores. With our small sample size, one outlier has a higher possibility of skewing results away from statistically significant correlation. Further research will be needed to determine if exclusion of this outlier was appropriate.

We did not study, nor do we advocate, any particular “pass” threshold for simulation evaluation. As in any other course, the instructor would need to establish this given the difficulty of content, ability of students to master material with the course format, and degree of “high stakes” activity.

The three components of the written testing have not been correlated with each other, as they are designed to test different cognitive skills. Therefore, correlation of their aggregate with simulation evaluation may lack a basic level of validation. Nevertheless, the simulation is new, labor intensive and expensive, and therefore more in need of scrutiny and validation. Our results of testing relatively novice learners may not be generalizable to more experienced providers. Lastly, the simulation evaluation raters had, at best, vague familiarity with the students. That they were identifiable on the recordings may have introduced an unknown bias into the evaluation.

## CONCLUSION

This study is the first to compare written and simulation-based evaluation in medical students. Simulation-based ACLS evaluation methods correlate with traditional written evaluation methods, and provide additional opportunity to demonstrate competency of resuscitation knowledge and skills.

Simulation may be a more discriminating and challenging testing method, as students scored higher on written evaluation methods compared to simulation. The meaning of this difference needs clarification through further research.

## Figures and Tables

**Figure:**
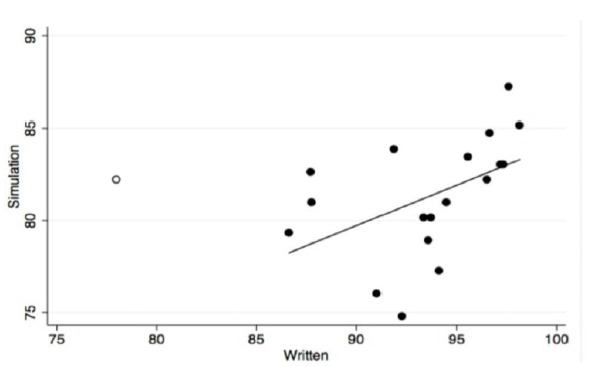
Correlation between mean percent correct score on traditional three-component written evaluation vs. percent correct score on simulation evaluation. Open circle student was excluded due to outlying low score on cardiac rhythm test.

**Table:** Individual and grouped percent correct performance scores for traditional written evaluation vs. simulation evaluation.

	Mean±SD
Multiple choice test	89.4±5.7%
Cardiac rhythm test	97.8±10.7%
Clinical management test	93.8±6.3%
Mean of three written tests	93.6±5.0%
Simulation test	81.3±3.2%
Difference	12.3±3.5%

*SD,* standard deviation
